# Objective structured clinical examination: Do first-year nursing students perceive it to be stressful?

**DOI:** 10.4102/curationis.v46i1.2339

**Published:** 2023-03-27

**Authors:** Dorothee Line Adibone Emebigwine, Ntombizodwa S.B. Linda, Penelope Martin

**Affiliations:** 1 School of Nursing, Faculty of Community and Health Sciences, University of the Western Cape, Cape Town, South Africa; 2 Department of Nursing, Faculty of Science, Agriculture and Engineering, University of Zululand, Empangeni, South Africa

**Keywords:** Clinical, first-year nursing students, Objective Structured Clinical Examination (OSCE), perception, stress

## Abstract

**Background:**

The Objective Structured Clinical Examination (OSCE) is widely accepted as an effective means of assessing clinical competence and nursing skills. There is, however, little existing knowledge on how first-year nursing students perceived stress during their first OSCE.

**Objectives:**

To determine the perception of stress; to identify the perceived factors causing stress; and to determine the perceived incidence of stress.

**Method:**

A descriptive, survey was conducted on a sample of 82 first-year nursing students using the Perceived Stress Scale (PPS).

**Results:**

The results showed that more than half (*n* = 54) of students perceived stress at moderate levels. Students not having sufficient time to complete the OSCE was perceived as the main factor causing stress (mean = 22.04; standard deviation [s.d.] = 6.21). The correlation between perception of stress and perceived factors causing stress showed a significant weak positive linear correlation among the variables (*r* = 0.45; *p* < 0.05).

**Conclusion:**

The study findings are important as the data determining the first-year nursing students’ perception of stress were collected immediately after their first OSCE, which may indicate that perception of stress was related to the actual event rather than the preparation for the OSCE. A follow-up qualitative research study should be conducted, preferably in the same setting, so that the students’ experiences of stress during the first OSCE can be explored in depth.

**Contribution:**

The OSCE environment is perceived as stressful for nursing students and will need careful support from academic and clinical teaching staff.

## Introduction

The effectiveness of the Objective Structured Clinical Examination (OSCE) at evaluating healthcare students’ multiple clinical skills and diverse subjects has widely been discussed (Al-Hamed 2021:186; Raziani, Nazari & Raziani 2022:1). Al-Hamed (2021:187) avows that for a final assessment, in relation to the traditional clinical assessment techniques, the OSCE offers a more unbiased, effective and dependable assessment technique. During the OSCE examination, students are observed while they demonstrate clinical behaviours or skills in standardised patient encounters (Al-Hamed 2021:188). However, Fawaz and Alsalamah (2021:6) acknowledge that while the OSCE can promote better performance and prepare students for the reality of clinical practice, it also induces stress for students. According to Lazarus and Folkman (1984), stress is defined as:

[*A*] particular relationship between the person and the environment that is appraised by the person as taxing and/or exceeding his or her resources and endangering his or her well-being. (p. 19)

Stress during the OSCE has been alluded to in existing literature. In a study reviewing papers across several countries (the United States [US], United Kingdom [UK], Jordan, Egypt, Saudi Arabia and Zambia) about the use of OSCE in nursing education and another study examining the OSCE Review perspectives of university nursing students and examiners to appraise necessary nursing skills, researchers found that a small amount of stress acts as a motivator, enhancing performance and encouraging the student to try harder (Al-Hamed 2021:186; Fawaz & Alsalamah 2021:6). On the other hand, excessive, undue and unnecessary stress was found to be disruptive to mental processes that are essential for students to perform well (Mojarrab, Bazrafkan & Jaberi 2020:5). In another study exploring nursing students’ perceptions and attitudes towards OSCE in Oman found that more than one-third of the participants (*n* = 22; 35%) declared that OSCE was stress-inducing and a difficult examination (Alamri et al. 2022:7). The reason cited was that it was a first-time experience for the respondents, as well as the first time the OSCE was implemented at the institution. Hence, it being a new experience, anxiety was inevitable.

The conceptual framework of the current study is based on the stress and coping theory of Lazarus and Folkman (1984:2005). It should be noted, however, that this study focuses on the description of first-year nursing students’ perceived stress and not the adaptation or coping process in nursing students, as is usual in studies that are framed by Lazarus and Folkman’s theory (1984:205). This theory was found to be a very useful means of identifying the presence or absence of stress experienced by the first-year nursing student during OSCE in the current study. According to Lazarus and Folkman (1984:205), a person can endure an overwhelming situation yet be able to acknowledge that the situation was stressful. Primary appraisal consists of a cognitive process in which a person asks, when encountering a stressful situation, questions like, ‘what does this stressor and/or situation mean?’ and ‘how can it influence me?’ According to Lazarus and Folkman, the three typical answers to these questions are ‘this is not important’, ‘this is good’ and ‘this is stressful’ (Lazarus & Folkman 1984:206).

Secondary appraisal comprises those feelings related to dealing with the stressor or generated stress (Lazarus & Folkman 1984:206). The person makes statements like ‘I can do it if I do my best’, ‘I will try whether my chances of success are high or not’ and ‘if this way fails, I can always try another method’, which indicate positive secondary appraisal (Lazarus & Folkman 1984:206). In contrast to these, statements like ‘I can’t do it, I know I will fail’, ‘I will not do it because no one believes I can’ and ‘I won’t try because my chances are low’ indicate negative secondary appraisal (Lazarus & Folkman 1984:206).

Primary appraisal suggests that the individual considers the stressor a threat that will cause harm, either in the present or in the future, such as failure in the OSCE (Lazarus & Folkman 1984:206). On the other hand, secondary appraisal occurs simultaneously with primary appraisal and is concerned with the feelings produced by the stressor or the stress produced (Lazarus & Folkman 1984:206). According to Lazarus and Folkman (1984:206), when the stressor is perceived as a challenge, a positive stress response is developed. An example of this is that if there is an expectation of a positive outcome, for example, passing the OSCE, the stress has a positive effect. Therefore, one can manage to endure an overwhelming situation yet be able to acknowledge that the situation was stressful. In line with Lazarus and Folkman’s study, the first-year nursing students appraised the OSCE as stressful and then decided how they could navigate through it.

At the institution where this study was conducted, the skills lab method was used to teach clinical skills to nursing students. The skills lab method has four phases which students must engage with to learn the clinical skill. These phases include visualisation, guided practice, independent practice and assessment (Jeggels, Traut & Kwast 2010:51). Teaching a nursing skill is well explained in the four phases of skills lab method. Given that students must practise skills that were demonstrated, opportunities to practise skills may be limited. This limited exposure may contribute to a lack of confidence in performing required nursing skills. While the experience of stress during assessments is documented in the literature, there is a paucity of literature on how first-year nursing students perceive stress during their first exposure to an OSCE. The aim thus of this study was to describe first-year nursing students’ perceived stress associated with their first experience of an OSCE at a University in the Western Cape. To achieve the aim, three objectives were formulated, namely (1) to determine the perception of stress, (2) to identify the perceived factors causing stress and (3) to determine the perceived incidence of stress.

## Research method and design

In this section, research and design and methods used in the study are discussed. A quantitative, descriptive survey design was used to achieve the aim of this study.

### Setting

This study was conducted at a School of Nursing at a residential university in the Western Cape, which is situated approximately 24 km from the Cape Town city centre. The School of Nursing offers two Bachelor of Nursing (BN) programmes, one which is studied over four years and an extended curriculum programme over a 5-year period. There were approximately 1200 nursing students studying in these two programmes at the time of data collection.

### Study population and sampling strategy

A total of 213 students registered for a Bachelors of Nursing programme in 2015 at the selected university for the first time constituted the population for the study. However, the targeted population was all first-year nursing students in the 4-year BN programme who qualified to sit for the Final Clinical Examination according to their Continuous Assessment Mark as per the university policy. Convenience sampling was used to select a sample first-time students who had obtained entry to the OSCE. Convenience sampling was used because it was imperative to use respondents who had never been exposed to the OSCE in academic life, which included their studies in other courses and/or in other institutions. Therefore, first-year nursing students were identified as a convenient group. Those students who were either repeating the first year or had been exposed previously were excluded from participation. All nursing students had to be exposed to the OSCE for the first time at the time of data collection.

### Data collection

The Perceived Stress Scale (PSS) developed by Cohen (1994:5) was used to collect data. The PPS is an existing, validated, 35-item, 5-point Likert-type structured questionnaire. The questionnaire had two sections: section A included demographics, comprising five questions, namely gender, age, marital status, place of residence and history of nursing studies. In section B, 30 items were categorised according to the three domains. Firstly, PSS – perception of stress included 10 questions which measured the degree to which the OSCE was appraised as stressful; secondly, a second category of item sought responses to ‘*Factors*’ causing stress, focusing on time and preparedness during the OSCE. This category would determine if the first-year nursing students perceived any factor as causing stress during the first OSCE. The following changes were affected by the researcher instead of asking ‘how often’ as the question are asked in the original questionnaire of Cohen (1994:5) the respondents were asked to respond to questions that phrased as ‘have you’. This reconstruction of the items changed the nature of the questions from being general statements to being statements specific to perceived factors causing stress during a first experience of the OSCE. Thirdly, the last category of question items addressed the ‘*Incidence*’ of stress relating to nursing students’ self-evaluation during the OSCE. In this section, the researcher focused on the intensity of the OSCE experience. The following change in the second category of the questionnaire was adapted by the researcher and the questions phrased as ‘have you’ instead of ‘how often’, as it was asked in the original questionnaire. This reconstruction of the questions changed the nature of the questions from requiring general information to being specific to the incidence of stress during a first experience of the OSCE.

Positively stated items (4, 5, 7, 8) from each domain were marked with an ‘*r*’ to indicate that their responses needed to be reversed (e.g. 0 = 4, 1 = 3, 2 = 2, 3 = 1 and 4 = 0). Data were analysed by scoring items in each domain from ‘0 to 4’; with ‘0’ indicating the absence of stress, ‘1’ indicating a low level of stress and ‘4’ indicating a high level of stress. The following scores were given: (1) a total scoring ranging from 0 to 13 was considered low stress; (2) scores ranging from 14 to 26 were considered moderate stress; and (3) scores ranging from 27 to 40 were considered high stress, as indicated by the PSS.

The questionnaire was pretested on three first-year nursing students. The adapted PSS used achieved a Cronbach’s alpha of 0.6, which is acceptable for an exploratory research study of this kind (Hair et al. 2006:504).

After each OSCE session, the students entered the venue (for data collection) in groups of eight, and they were encouraged to sit and relax for a few minutes before signing the consent forms. The potential respondents were given a chance to ask questions to clarify any aspect that was not fully clear to them or raise any concerns relating to the questionnaire. The completed consent forms were inserted into a self-sealing, opaque envelope and placed in sealed box marked ‘consent forms’. Potential participants were also assured that their participation or nonparticipation would not influence their OSCE marks in any way. Thereafter, each respondent was provided with a questionnaire and requested to put the completed questionnaire in a sealed box marked ‘questionnaires’ once they had completed them. Respondents completed the questionnaires in the presence of the researcher.

### Data analysis

Data were analysed using Statistica version 13 (TIBCO Software Inc., Palo Alto, California, United States). Descriptive statistics were used to analyse and describe first-year student nurses’ perceptions of stress during their first OSCE. The Kruskal–Wallis analysis of variance (ANOVA) or one-way ANOVA was used to determine whether there was a significant difference between two groups, for example, male and female, a perception of stress during the OSCE and/or an incidence of stress among male and female participants during the OSCE.

### Ethical considerations

Ethical clearance to conduct this study was obtained from the University of the Western Cape Senate Research Committee (ref. no. 15/4/56) and formal permission to approach the students was obtained from the Head of the School of Nursing. Participation in this study was purely voluntary. All participants, before filling the questionnaires, had to sign the consent form for their participation. Confidentiality was ensured by promotion of anonymity by not requiring respondents to write their names on their questionnaires. All completed questionnaires were dropped in a sealed box by the respondent. This box was accessed by the researcher only, and where necessary, the contained information was shared with the supervisor, co-supervisor and the statistician.

## Results

A total of 82 respondents participated in this study, yielding a response rate of 38%. The demographic characteristics of the participants in this study are displayed in Table 1. Most of the respondents (78%, *n* = 64, standard deviation [s.d.] = 5.14) were female; less than a quarter (22%, *n* = 18) were male. Less than half (43.9%, *n* = 36) of the respondents were between 18 and 20 years of age. Slightly more than a third (35.3%, *n* = 29) of the respondents were young adults aged between 21 and 34 years.

**TABLE 1:** Respondents’ demographic characteristics.

Variable	Frequency	%
**Gender**		
Male	18	22
Female	64	78
**Age**		
18–20	36	43.9
21–34	29	35.3
35–45	4	4.8
No response	13	15.8
**Marital status**		
Married	5	6.0
Single	76	93.0
Separated	1	1.0
**Nursing as first postmatric or tertiary education?**		
Yes	65	79.0
No	17	21.0

Students’ perceived stress ranged from 0.6 and 3.5, with a mean of 2.14 (s.d. = 0.55); however, there were no significant gender differences in terms of perceived stress.

The majority 66% (*n* = 54) of students perceived a moderate level of stress during their first OSCE (Table 2), with a mean PSS score of 2.14 (± 0.55) (Table 3), while 32% (*n* = 26) of students perceived a low level of stress during their first OSCE and 2% (*n* = 2) of students perceived a high level of stress during their first OSCE.

**TABLE 2:** Level of stress for perception of stress.

Level of stress	Numbers	%
High stress	2	2
Moderate stress	54	66
Low stress	26	32

**TABLE 3:** Mean of nursing students’ perception of stress.

Scale	*N*	Mean	Range		s.d.
			Minimum	Maximum	
Perception of stress	82	2.14	0.6	3.5	0.55

s.d., standard deviation.

Students’ perceived factors causing stress ranged between 0.9 and 3.5, with a mean of 2.36 (s.d. = 0.67). Students not having sufficient time to complete the OSCE was perceived as the main factor causing stress (mean = 22.04; s.d. = 6.21), followed by stress related to long waiting time in the corridor for the OSCE (mean = 22.03; s.d. = 6.06) and stress from having to perform in front of an evaluator during the OSCE (mean = 21.69; s.d. = 6.03). The lowest source of stress was from students’ feeling of lack of preparation before the OSCE (mean = 20.53; s.d. = 6.65). However, there were no significant gender differences in terms of perceived factors causing stress.

The majority of students (93%, *n* = 75) perceived factors causing stress at moderate levels (Table 4) with a mean Factors Causing Stress Scale score of 2.36 (± 0.67) (Table 5). However, 2% (*n* = 2) of students perceived factors causing stress at a high level, and 5% (*n* = 4) of students perceived factors causing stress at a low level.

**TABLE 4:** Level of stress for factors causing stress.

Level of stress	Numbers	%
High level of stress	2	2
Moderate level of stress	75	93
Low level of stress	4	5

**TABLE 5:** Mean of factors causing stress.

Scale	*N*	Mean	Range		s.d.
			Minimum	Maximum	
Factors causing stress	82	2.36	0.9	3.5	0.67

s.d., standard deviation.

The mean students’ perceived incidence of stress was 2.21 (s.d. = 0.62) and ranged from 0.8 and 3.8. There are statistically significant differences between female (mean = 2.29; s.d. = 0.59) and male students (mean = 1.90; s.d. = 0.64) in their incidence of stress (*t* = 0.02; *p* < 0.05).

The majority of students (93%, *n* = 74) perceived incidence of stress at a moderate level (Table 6), with a mean Incidence of Stress Scale score of 2.21 (± 0.62) (Table 7). However, 4% (*n* = 3) perceived incidence of stress at a high level, while 4% (*n* = 3) perceived incidence of stress at a low level during the first OSCE. Students who perceived the incidence of stress at a low level had a significant positive correlation than those who perceived the incidence of stress at a moderate level (*r* = 0.01; *p* < 0.05).

**TABLE 6:** Level of stress for incidence of stress.

Level of stress	Numbers	%
High level stress	3	4
Moderate level stress	74	93
Low level of stress	3	4

**TABLE 7:** Mean of nursing students’ incidence of stress.

Scale	*N*	Mean	Range		s.d.
			Minimum	Maximum	
Incidence of stress	82	2.21	0.8	3.8	0.62

s.d., standard deviation.

The correlation between perception of stress and perceived factors causing stress showed a significant weak positive linear correlation among the variables (*r* = 0.45; *p* < 0.05); in addition, the correlation between perception of stress and the perceived incidence of stress showed a significant weak positive linear correlation among the variables (*r* = 0.36; *p* < 0.05). However, the correlation between the perceived factors causing stress and the perceived incidence of stress showed a moderate positive linear correlation among the variables (*r* = 0.64; *p* < 0.05).

## Discussion

The literature suggests that OSCE is associated with high levels of stress and anxiety (Wadi et al. 2022:54; Wu et al. 2020:854). In this study, although the number of female students was considerably higher than male students, there were no significant gender differences in terms of perceived stress, perceived factors causing stress and perceived incidence of stress. These findings are consistent with the literature and across various disciplines (dental students, medical students). In a study that examines the effects of anxiety on dental students in their first OSCE, although the majority of participants were male, the authors found that female students presented higher anxiety levels than male students; however, there was no statistical gender difference in students (Arain 2021:104; Wadi et al. 2022:52; Wu et al. 2020:854). In the current study, the demographic characteristics age, marital status and nursing as the first tertiary education did not yield any significant differences in terms of perceived stress, perceived factors causing stress and perceived incidence of stress. These findings are consistent with previous studies (García-Mayor et al. 2021:3; Mojarrab et al. 2020:4; Wadi et al. 2022:52; Wu et al. 2020:854).

Several studies conducted on dental, medical and nursing students have found the OSCE considerably more stressful than other forms of assessments (Alamri et al. 2022:8; Raziani et al. 2022:5; Vincent et al. 2022:1). It causes confusion, and distraction reduces the academic performance (Fawaz & Alsalamah 2021:6; Raziani et al. 2022:5). In the current study, the majority of students perceived stress at a moderate level during their first OSCE. These results are consistent with previous studies conducted among nursing students within diverse nations. In Iran, a study comparing test anxiety in OSCEs and traditional assessment methods among undergraduate midwifery students found that 56.9% of the students experienced moderate test anxiety (Faramarzi et al. 2013:2206). Similarly, in Taiwan, a study that explored the effects of anxiety on dental students in their first OSCE found that the majority of participants exhibited moderate levels of both state and trait anxiety in their first OSCE (78.8% and 79.2%, respectively) (Wu et al. 2020:854). In a review of nursing faculty and students’ perceptions and experiences that compare OSCE and traditional clinical examination to students’ clinical competence, the reviewer found that the mean of moderate-to-severe test anxiety was more in traditional clinical examinations than with OSCE (Vincent et al. 2022:11). In a study that explored the experiences of undergraduate nursing students and examiners with the OSCE as an evaluation of physical assessment skills, the participants perceived the exam to be a stressful experience (Bani-Issa et al. 2019:86).

In the current study, it is interesting that the majority of students perceived factors causing stress at a moderate level during their first OSCE. This was an unexpected finding, because in the faculty under study, students are well prepared for the OSCE by means of a mock OSCE which emulates the real situation. In a mixed-methods study by Bani-Issa et al. (2019:86), students felt that it would be helpful to have mock exams before the real test. One student commented:

‘It is good to have “prova” before the real OSCE, this will improve our grade in the exam and will take off the fear and stress we had during the OSCE.’ (Participant 1)

Furthermore, the perceived factors causing stress for the students during their first OSCE were: students not having sufficient time to complete their OSCE, stress related to long waiting time in the corridor for their OSCE and stress related to having to perform in front of an evaluator during their OSCE. This is congruent with previous studies. In a study conducted in Oman, the participants stated that the time allocated to read the OSCE instructions and the time allocated to complete the procedure were insufficient (Alamri et al. 2022:8; Bani-Issa et al. 2019:86; Fawaz & Alsalamah 2021:3; Majumder et al. 2019:390). In another study conducted in Spain, the researchers found that the students displayed pre-emptive anxiety before the exam in anticipation of the unfamiliar event (Sánchez-Conde, Beltrán-Velasco & Clemente-Suárez 2022:5). Finally, in other previous studies, the participants stated that performing in front of unfriendly examiners and in front of other students was stress-inducing (Alamri et al. 2022:8; García-Mayor et al. 2021:4; Raziani et al. 2022:3).

Literature found that students experienced stress in anticipation of the upcoming OSCE while in the waiting area, as well as while progressing through the OSCE and after the OSCE (Mojarrab et al. 2020:4; Sánchez-Conde et al. 2022:5). These findings are congruent with the current study that showed the majority of the respondents’ perceived incidence of stress at a moderate level.

During the skills lab sessions, students are taught using the most common cases and procedures which they should master in preparation for the OSCE (Jeggels et al. 2010:51). In this study, however, a weak positive linear correlation was found between perception of stress and the perceived factors causing stress; between perception of stress and the perceived incidence of stress; and a moderate positive linear correlation between factors causing stress and the perceived incidence of stress. These showed that although the students experience stress during their first OSCE, the stress had a positive impact in their performance during the OSCE (Alamri et al. 2022:8; Raziani et al. 2022:5). This suggests that there is positive stress that helps students achieve good results, and there is negative stress that bears bad results (Mojarrab et al. 2020:5). The OSCE has been found to be beneficial for students, as it is associated with the sense of achievement which prepares students for their subsequent nursing clinical placement (Alamri et al. 2022:8). Furthermore, it increases the confidence of students in their learning ability by helping them to identify areas of weakness and gaps in their competencies (Alamri et al. 2022:8; Bani-Issa et al. 2019:85). In a mixed-methods study by Bani-Issa et al. (2019:86), most students perceived the OSCE exam to have good qualities and objectives. One student commented:

‘[*I*]t was a fair exam … I know that I’m weak in differentiating respiratory sounds … the exam detected my deficit … now I need to do practice on that skill.’ (Participant 2)

Despite the potential beneficial aspect of the stress during OSCE, however, such disturbances may affect the psychological or emotional well-being and the professional life of these students and ultimately affect the quality of patient care they provide (Sánchez-Conde et al. 2022:5).

### Limitations

The results of this study cannot be generalised because of the small sample; nevertheless, the results of this study will increase the body of knowledge about first-year nursing students’ perception of stress during the first OSCE. Another potential limitation of this study is that the data were collected just after the first-year students’ first OSCE had been conducted as an aspect of their final examination. The students were asked to complete the questionnaire immediately after they had completed the OSCE. The rationale to collect data immediately after OSCE was based on the assumption that it was better to complete the questionnaire while the experience of the phenomenon under inquiry was still fresh in their minds. However, the students appeared to have been eager to leave the examination area as soon as they possibly could. This may partly explain why a great number of students did not participate in the study. Had the students been asked to complete the same questions hours or days later when there was little or no stress, a greater number of students may have taken part in the study.

### Recommendations

The findings of the study indicated the following recommendations which were strongly advocated by the participants: *firstly*, the participants of the current study indicated the need to use the OSCE for clinical practice throughout the year and before their final assessment of clinical skills. They believe that it could help to reduce stress during the actual final OSCE at the end of the year. *Secondly,* collaboration with clinical practice was suggested to be conducted during practice sessions in real-life settings at the centre of students’ learning, thus keeping the live-simulation learning and assessment strategy as free from stress as possible.

## Conclusion

The results of the study indicated that first-year nursing students perceived moderate stress during their first OSCE. Despite the existing difficulty in producing a simulated environment within clinical skills laboratory settings that resemble the complexity of a clinical area, the OSCE environment remains stressful for nursing students. Some participants in this study also felt anxious, angry and uncomfortable during the OSCE. These students need careful support from academic and clinical teaching staff.
